# Photodynamic Therapy for Pancreatic Ductal Adenocarcinoma

**DOI:** 10.3390/cancers13174354

**Published:** 2021-08-28

**Authors:** Vida Karimnia, Frank J. Slack, Jonathan P. Celli

**Affiliations:** 1 Department of Physics, University of Massachusetts at Boston, Boston, MA 02125, USA; vida.karimnia001@umb.edu; 2 Department of Pathology, BIDMC Cancer Center/Harvard Medical School, Boston, MA 02215, USA; fslack@bidmc.harvard.edu

**Keywords:** photodynamic therapy (PDT), photomedicine, pancreatic ductal adenocarcinoma (PDAC), pancreatic cancer, stroma, combination therapy, drug delivery

## Abstract

**Simple Summary:**

Pancreatic ductal adenocarcinoma (PDAC) is among the most lethal of human cancers. Numerous clinical trials evaluating various combinations of chemotherapy and targeted agents and radiotherapy have failed to provide meaningful improvements in survival. A growing number of studies however have indicated that photodynamic therapy (PDT) may be a viable approach for treatment of some pancreatic tumors. PDT, which uses light to activate a photosensitizing agent in target tissue, has seen widespread adoption primarily for dermatological and other applications where superficial light delivery is relatively straightforward. Advances in fiber optic light delivery and dosimetry however have been leveraged to enable PDT even for challenging internal sites, including the pancreas. The aim of this article is to help inform future directions by reviewing relevant literature on the basic science, current clinical status, and potential challenges in the development of PDT as a treatment for PDAC.

**Abstract:**

Pancreatic ductal adenocarcinoma (PDAC) is among the most lethal of human cancers. Clinical trials of various chemotherapy, radiotherapy, targeted agents and combination strategies have generally failed to provide meaningful improvement in survival for patients with unresectable disease. Photodynamic therapy (PDT) is a photochemistry-based approach that enables selective cell killing using tumor-localizing agents activated by visible or near-infrared light. In recent years, clinical studies have demonstrated the technical feasibility of PDT for patients with locally advanced PDAC while a growing body of preclinical literature has shown that PDT can overcome drug resistance and target problematic and aggressive disease. Emerging evidence also suggests the ability of PDT to target PDAC stroma, which is known to act as both a barrier to drug delivery and a tumor-promoting signaling partner. Here, we review the literature which indicates an emergent role of PDT in clinical management of PDAC, including the potential for combination with other targeted agents and RNA medicine.

## 1. Introduction

Pancreatic ductal adenocarcinoma (PDAC) is among the most lethal of human malignancies, with a 5-year survival rate of approximately 10% in the USA [1,2]. Surgery is possible in only 20% to 30% of patients and options are particularly limited for patients with unresectable disease [3]. Where resection is possible, the Whipple procedure, or pancreaticoduodenectomy is performed, in which the head of the pancreas, duodenum, gallbladder, and the bile duct are removed. This is a complex procedure with a significant impact on quality of life [4,5]. Virtually all clinical trials of chemotherapy drugs, targeted agents and combinations have failed to provide meaningful improvements in survival and most patients ultimately end up receiving palliative treatment [6,7].

For patients with advanced disease, palliative treatment has traditionally involved chemotherapy with either 5-fluorouracil (5-FU) or gemcitabine. In a clinical study reported in 1997, gemcitabine was found to impart a slight survival advantage (median = 5.65 months) compared with 5-FU (median = 4.41 months) and modest improvement in quality of life [8]. Since then, gemcitabine has become a mainstay for palliative care of advanced PDAC. More recently, a multidrug cocktail called FOLFIRINOX has been used, which is the combination of 5-FU with three other chemotherapy regimens (oxaliplatin, irinotecan, and leucovorin). Compared to gemcitabine, the FOLFIRINOX regimen achieves significant benefit in median survival of 11.1 months vs. 6.8 months for gemcitabine, though with significantly increased toxic effects, making it a viable option only for patients who are otherwise relatively healthy [9]. In a later study, survival benefit was observed to be further enhanced using a modified FOLFIRINOX combination [10]. Collectively, these results point to the fact that there remains an urgent need for strategies to overcome this disease.

**Figure 1:**
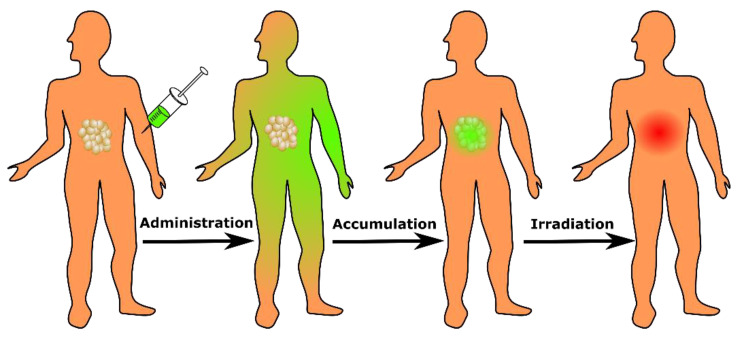
PDT workflow schematic. Following PS administration, there is a delay period during which the PS accumulates in malignant tissue, followed by light activation at the target site.

This review focuses on the clinical and preclinical literature which explores the potential role of photodynamic therapy (PDT) in clinical management of PDAC. PDT is a photochemistry-based modality that selectively destroys target tissue by exciting a photosensitizer (PS) with light of an appropriate wavelength. The PS is typically administered intravenously followed by a delay period to allow for accumulation in the tumor prior to irradiation using a light delivery system appropriate for the target tissue. While the PS itself accumulates preferentially in malignant tissues, an additional degree of selectivity is achieved by directing light to target tissue (Figure 1) [11]. The exact manner of light delivery is highly dependent on the anatomical site of treatment. For treatment of pancreatic cancer, laser light delivery is achieved through interstitial optical fibers placed directly into the lesion under either CT guidance or endoscopically ultrasound [12,13] as discussed in more detail further below.

PDT has been approved by the United States Food and Drug Administration (FDA) to treat patients with non-small cell lung cancer, esophageal cancer, and Actinic Keratoses, as well as age-related macular degeneration [14,15,16]. Clinical trials have also demonstrated PDT efficacy for mesothelioma, prostate, bladder, brain cancer, and head and neck cancers as well as bacterial, fungal, and viral infections [16,17,18,19]. In many cases, photosensitizing agents are also used off label with noted successes by clinicians who are comfortable with photomedicine and laser light delivery. In this review, we focus specifically on the preclinical and clinical status of PDT for treatment of pancreatic cancer, a lethal disease which presents multiple challenges for treatment by any modality. We will specifically examine how the biology of pancreatic cancer, which is characterized by prominent stromal involvement, presents challenges for drug delivery, and implications and opportunities for PDT.

## 2. PDT Mechanism and Clinical Implementation

**Figure 2:**
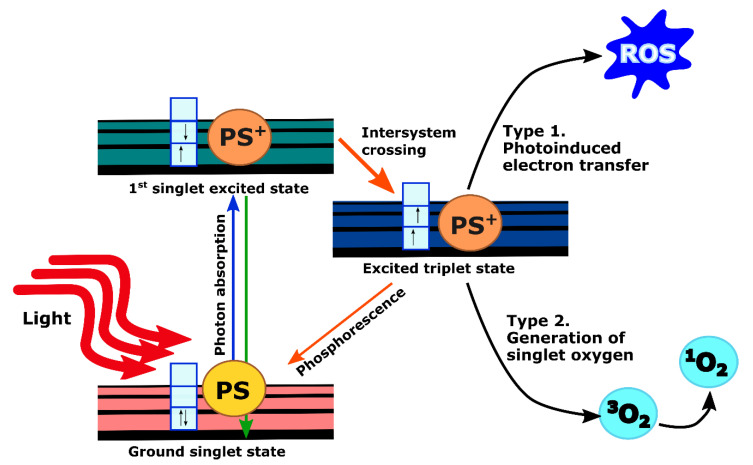
Photophysics and photochemistry of PDT. Vertical arrows in boxes indicate electron spin states.

When PS molecules absorb light, they undergo excitation from the ground state to an excited state depending on incident photon wavelength. Excited molecules rapidly drop back to their lowest vibrational level of the electronic excited state from which they can return back to the ground state either through non-radiative decay or by emitting photons with longer wavelength and lower energy (fluorescence). These processes have a short lifetime (in the order of nanoseconds) and do not lead to subsequent photochemistry. However, fluorescence emission from the PS, as a tumor-localizing fluorophore, is invaluable for imaging purposes as reviewed extensively elsewhere [20]. Another possibility is that the excited PS molecules undergo intersystem crossing to an excited triplet state. The lifetime of these states is long (in the order of microseconds or milliseconds) since the spin states are parallel instead of anti-parallel. Thus, it is forbidden for the PS molecules to go back to the ground state. Instead, they could either initiate photochemical reactions by transferring electrons to form reactive oxygen species (ROS) (type 1), or transfer their energy to the ground-state triplet oxygen molecule (^3^O_2_) to give rise to singlet oxygen molecule (^1^O_2_) through collisional quenching (type 2). These products are highly reactive and can cause cellular toxicity (Figure 2) [20,21]. Although type 1 PSs are effective even in a hypoxic environment, all current clinically approved PSs, including those which have been studied for pancreatic cancer, impart toxicity primarily by the type 2 mechanism [22].

PDT does offer several inherent advantages. Depending on the localization of the PS, PDT can directly damage or alter targets in tumor cells. Additionally, since the visible or near infrared light used in PDT is non-ionizing, PDT does not carry the accumulating toxicity associated with radiotherapy [12,23]. However, unlike ionizing radiation, a noted challenge with PDT is the limited penetration of red and near-infrared wavelengths in tissue. Light delivery for internal sites such as the pancreas requires careful treatment planning and dosimetry, though innovative solutions have been developed and clinically validated as discussed further below.

## 3. PDT for Pancreatic Cancer: Early Preclinical Development

PDT for pancreatic cancer has been evaluated using a wide variety of preclinical models and photosensitizing agents. The first-generation PSs tested on pancreatic cancer were simple organic molecules with moderate inherent selectivity for neoplastic tissues. Several PS agents such as hematoporphyrin derivative (HpD), dihematoporphyrin ether (DHE), and Photofrin had a disadvantage of causing skin photosensitivity of up to 2–3 months [24]. Moreover, the necrosis produced in pancreatic cancer in some animal studies (rodents) using DHE, pheophorbide A, and aluminum-sulphonated phthalocyanine (AlSPc) caused notable complications such as duodenal perforation due to significant PS accumulation in the surrounding tissue. However, using a lower dose of PS markedly reduced damage. This could be avoided by shielding duodenum during light exposure, and also it was considered less likely to be problematic in human duodenum which is much thicker than the duodenum in animal models used in studies [24,25,26,27]. This work generally underscored the importance of selection of PS and light delivery strategy. As discussed further below, these early preclinical studies paved the way for clinical work in addition to wide-ranging and ongoing preclinical investigation of PDAC response to PDT and PDT combinations with other treatments for PDAC.

## 4. Clinical PDT for Pancreatic Cancer: Treatment Planning, Guidance and Monitoring

**Figure 3:**
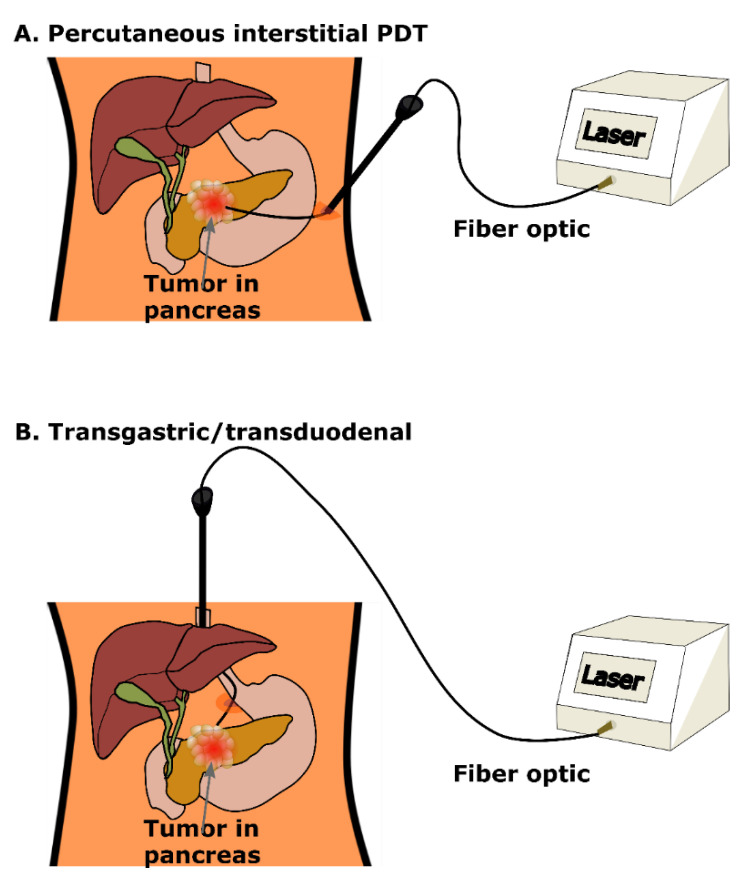
Percutaneous interstitial PDT.

Over the course of the past 20 years, there has been significant advancement in clinical use of PDT for treatment of pancreatic cancer using different photosensitizers and strategies to deliver light to the pancreas. A pilot clinical study of PDT for pancreatic cancer was conducted by Bown et al. in 2002, on 16 patients using mesotetrahydroxyphenylchlorin (mTHPC) [12]. To overcome the limitation of light attenuation in tissue, the light was delivered using fiber optics positioned under computerized tomographic guidance (Figure 3A). The result showed substantial tumor necrosis. The median survival time after PDT was 9.5 months and there was no treatment-related mortality. A more recent phase I/II clinical study has successfully established the safety and technical feasibility of PDT for locally advanced PDAC using verteporfin [28]. This study was comprised of two phases. The first phase was dedicated to establishing the zone of necrosis based on the delivered light dose to choose the best light dose for the second part of the study which utilized multiple fibers. With the chosen dose of verteporfin of 0.4 mg kg^−1^, the goal of the first phase was to acquire a light dose to produce a zone of necrosis of at least 12 mm in diameter using just a single fiber. They started with an initial light dose of 5 J cm^−1^ and doubled it as long as there was no evidence of toxicity in any of the patients. The results indicate that a 12 mm necrosis can be obtained at 40 J cm^−1^ irradiation. In the second phase of the experiment, the light was delivered directly to the tumor via multiple fiber optics with 1–2 cm long diffuser tips positioned subcutaneously under CT guidance. The 690 nm laser was calibrated to deliver 5 J cm^−1^ along each diffuser tip of the fiber. Moreover, although some patients who were treated with a single fiber experienced some mild to moderate complications after PDT such as abdominal pain, transient rise in amylase, and diarrhea, there was no severe PDT-related complications. In fact, no patient showed any problems with photosensitivity and no evidence of early duodenal obstruction was reported. The patients who were treated with multiple fibers, however, showed evidence of inflammatory change along the needle tract. The investigations showed that although the light intensity in those regions was significantly less than the emission zones, the overlapping fields due to using multiple fibers resulted in those areas receiving a higher dose than the patients treated with a single fiber. Furthermore, the advantages of using verteporfin photosensitizer over mTHPC according to this study were verteporfin’s rapid excretion (peak tissue concentration within an hour or two), and its strong absorption at a 690 nm wavelength at which light can penetrate deep into the tissue.

In planning PDT treatments, accurate dosimetry is a key consideration. Additionally, in the case of PDT, dosimetry can be challenging, involving complex interactions between light (and light penetration through tissue) and local concentrations of photosensitizer and oxygen, which are key determinants of clinical PDT efficacy. Treatment planning typically requires a combination of accurate clinical measurement of these components, combined with simulation [29]. In a PDAC clinical trial referenced above, pre-treatment contrast CT images were used to analyze contrast difference values and ultimately, analyze the PDT-induced lesion volume [28]. Generally, the dosimetry measurements can be categorized as either explicit, implicit, or surrogate dosimetry (using a borrowed dosimetry marker) [29,30]. In explicit dosimetry, the main components of the photodynamic reaction (light, photosensitizer, and oxygen) are measured directly and incorporated into a dose model. Implicit dosimetry takes into account the non-measurable effects for contributing to the dose delivery such as photobleaching [31]. In surrogate dosimetry, some markers will be borrowed by standard clinical practice. As an example, the amount of contrast uptake in CT could predict PDT efficacy. As a result, it could be considered as a surrogate dosimetry measurement to prescribe light doses based on the pre-treatment contrast.

Other studies have taken advantage of the proximity of the stomach as a route for endoscopy to the pancreas for endoscopic ultrasound (EUS)-guided PDT [32]. In this method, after injecting porfimer sodium, a small diameter fiber with a cylindrical light diffuser is passed through the EUS needle and used to illuminate the tissue with laser light (Figure 3B). Choi et al. reported the first clinical study of EUS-PDT in pancreatic cancer. The median volume of necrosis produced by PDT in that study was 4.0 cm^3^ [33]. A recent phase I clinical study of porfimer sodium-mediated EUS-PDT followed by nab-paclitaxel and gemcitabine chemotherapy on patients with locally advanced pancreatic cancer further demonstrated the safety and feasibility of this method [34]. Although effective, porfimer sodium has a long half-life on the scale of days which leads to prolonged duration of cutaneous photosensitivity post procedure. The treatment resulted in a median of 2.6 months progression-free survival time. The study further showed that the chemotherapy after EUS-PDT may lead to the tumor downstaging and ultimately permit attempted surgical resection. Benefitting from the short half-life of verteporfin on the scale of hours, and the fact that it is a United States Food and Drug Administration-approved second-generation photosensitizer while offering a significant patient safety advantage, a clinical study published in 2021 evaluated the efficacy of verteporfin-mediated PDT administered under EUS guidance in patients with locally advanced pancreatic cancer [13]. This pilot study was performed on eight patients in stages 1–3. The treatment resulted in the tumor necrosis zone being visible on CT after 48 h in the majority of patients as well as lower indices of sinistral portal hypertension and arterial vascular involvement. The study concluded that EUS-guided, verteporfin-mediated PDT is a safe and promising therapy to enhance tumor response in selective patients with pancreatic cancer who were not responsive to chemotherapy.

Collectively, these studies indicate the feasibility and safety of the clinical use of PDT for pancreatic cancer. Additional data from a larger group study with a variety of conditions such as different energy doses, which were not used in these studies, will help solidify the optimal patient–procedure factors.

## 5. Role of PDAC Stroma and Implications for PDT

PDAC is characterized by the development of a particularly dense fibrotic stroma, including cellular and non-cellular components such as pancreatic stellate cells (PSCs), which differentiate into heterogeneous fibroblastic cells, type I collagen, immune cells, adipocytes, and hyaluronan [35]. This complex microenvironment plays multiple roles in regulating tumor growth and response to therapy, and as discussed here, presents challenges and opportunities for PDT.

**Figure 4:**
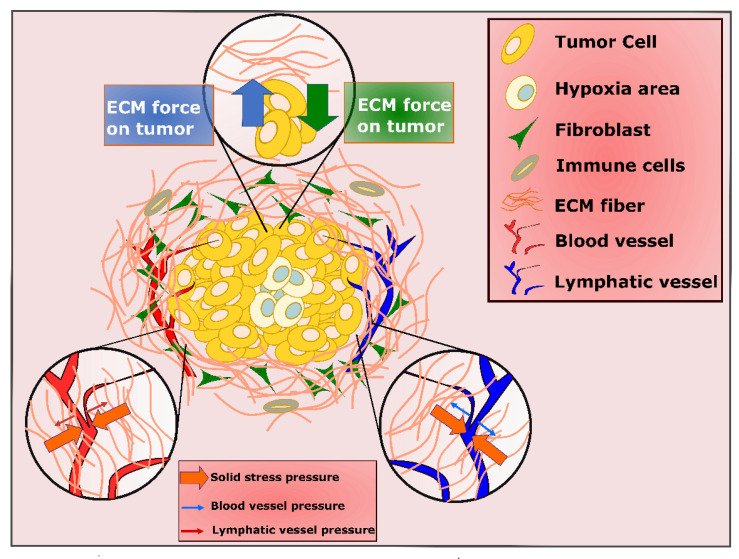
The biophysical tumor microenvironment.

One important consequence of the profound desmoplastic reaction in PDAC is the impact of compressive stress from accumulated fibrotic stroma, not only on cancer cells, but on blood and lymphatic vessels as well (Figure 4) [36]. The ill-functioning blood and lymphatic vessels limit drainage out of the tumor, causing elevated interstitial fluid pressure (IFP) [37,38,39,40]. As IFP increases to the value of microvascular pressure (MVP), the transportation of molecules to the tumor stops, which can lead to impermeability of large parts of PDAC tumor to therapeutic deliveries [41]. These hypo vascular tumors are also highly hypoxic, with oxygen percentage dropping from 7.5%, which is the estimated level in normal pancreas, to 0.3% in the pancreatic tumor [42]. While targeting tumor vasculature on one hand is a target for therapy, when vasculature is destroyed to cut off nutrition in cancer cells, it can also cause hypoxia through stimulation of several signaling pathways [43,44,45,46,47,48]. The role of hypoxia is further complicated in PDT, which requires oxygen and can also exacerbate hypoxia in tumors by consuming oxygen which is already present in order to produce ROS.

The challenge of tumor hypoxia has motivated efforts to design oxygen delivery/producing strategies to enhance PDT response in PDAC. As an example, there have been many studies utilizing oxygen-loaded microbubbles to deliver oxygen to the tumor microenvironment [49,50,51]. Additionally, since as cells progress toward malignancy, they become capable of producing excessive amount of H_2_O_2_, some studies have been focusing on in situ oxygen production by reaction between H_2_O_2_ and nanoparticles, nanorods, or catalase [52,53,54].

The profound and stiff stroma in PDAC not only exacerbates PS and oxygen delivery, but it also can reduce any drug delivery to the tumor. The first approach to improve drug delivery was to therapeutically target stroma components. This approach later emerged as a double-edged sword since although removing stroma increased drug delivery to the tumor, it also eliminated the physical and biochemical barriers that inhibited tumor progression [55]. Another approach has been using angiotensin receptor blockers (ABRs) which downregulate TGF-β; thus, inhibiting collagen production [37,56]. Moreover, targeting discoidin domain receptor 1 and 2 (DDR1 and DDR2) which is a pathway for promoting tumor progression, in parallel with conventional chemotherapy, has the potential to improve the outcome of PDAC treatment [57]. Sonic hedgehog (Shh) signaling is another important factor in developing PDAC [58]. Preclinical mouse model of pancreatic cancer studies demonstrated better drug delivery after inhibiting the Shh pathway [59]. Although this approach also showed promise in a phase I clinical trial [60], it failed in phase II [61]. Furthermore, a later study demonstrated that Shh inhibition led to acceleration of PDAC progression [62]. Another approach for improving drug delivery and hypoxia has been remodeling PDAC blood vessels by transforming the growth factor-β (TGF-β) signaling pathway which is involved in tumor vascular endothelial cells’ adhesion to pericytes (capillaries) [63,64]. Additionally, the heteroaromatic rings in the PSs make them prone to aggregation, thereby limiting ROS production, and decreasing phototoxicity [65,66]. Some innovative strategies have overcome this challenge as well by encapsulating PSs in nano carriers. This can facilitate the PSs delivery to the tumor or targeted stromal cells without self-quenching [67,68,69]. The role of PDT in this context is discussed later in this review in emerging and future directions section.

The cross talk between PDAC cells and the prominent cellular components and the mechanical interactions between extracellular matrix and PDAC have vital roles in tumor growth, invasion, and epithelial–mesenchymal transition (EMT) [70,71,72]. Pancreatic stellate cells (PSCs) are a key player in the PDAC tumor microenvironment [73]. In normal pancreas, PSCs generally remain in a quiescent phenotype. In PDAC, the quiescent PSC can adopt an activated phenotype with dramatically altered function as participants in paracrine tumor–stroma crosstalk. Stimulation via TGF-β and EGF activates PSCs promoting proliferation, migration, and production of laminin, fibronectin, collagen type I and III, contributing to activation of invasive behavior which is marked by downregulation of E-cadherin and upregulation of N-cadherin, consistent with increased EMT and the adoption of a more motile phenotype [74,75].

Although the role of interactions between PDAC cells and activated PSCs, which through differentiation, are the major source of cancer-associated fibroblasts (CAFs) in PDAC, has been extensively studied, a complete understanding of CAF heterogeneity and the roles that different CAF subtypes play is still emerging. Recently, studies have focused on the co-existence of two distinct subtypes of CAFs in PDAC: CAFs that express a high level of α-SMA which has been implicated with a tumor-suppressive role and is located primarily adjacent to cancer cells (myCAF), and CAFs that express high levels of cytokines and chemokines, associated with tumor-promoting behavior, and are located farther away from the cancer cells (iCAFs) [76]. An in vivo study of single-cell RNA sequencing on PDAC confirmed the existence of myCAF and iCAF subtypes and identified a new CAF subtype called antigen-presenting CAFs (apCAF) that can activate CD4^+^ T cells in an antigen-dependent manner [77]. Our previous study showed that while PDT response was enhanced in the presence of two fibroblastic phenotypes (myCAFs and iCAFs), the effect was more significant in cocultures that gave rise to iCAF phenotype, which were also the most chemoresistant [78].

The overexpression and accumulation of ECM proteins in PDAC contributes to the development of a stiff fibrotic stroma, which leads to not only physically restricting drug penetration, but also abnormal mechanotransduction in cancer cells [72,79]. Integrins constitute a superfamily of 24 heterodimeric cell surface receptors, and play a vital role in transducing mechanical signals between the intracellular and extracellular components [80]. Given that integrins regulate cellular proliferation, adhesion, invasion, and cancer progression, it is not surprising that some integrins have been used as potential therapeutic targets in PDAC [81,82,83,84]. While there have been some studies that focused on the impact of PDT on integrins in different types of cancers, there have not been any studies that investigated targeting integrins with PDT in PDAC [85,86,87].

Moreover, it is important to know that both biophysical and biochemical properties ultimately impact on the fate of the tumor. Although the stiffness associated with PDAC desmoplasia can promote invasion and malignancy, biochemical interactions between ECM and PDAC cells can also regulate tumor growth and cell invasion [88,89,90,91]. For example, in 3D cell culture models, increased PDAC invasion has been observed in a softer environment (rich in collagen I) compared to a stiffer laminin-rich environment, underscoring the importance of both biochemical and mechanical properties of the ECM [92,93]. There is also a distinction between the activation of invasive behavior (which can be promoted by confinement and stiffness of surrounding material) and invasion itself, which requires enzymatic degradation (and hence softening) of ECM to enable invasive motility. Notably, in the same study, it was shown that populations of drug-resistant cells with increased invasiveness correlated with increased EMT were also more responsive to PDT [92]. This is consistent with a previous report which also showed increased EMT in drug-resistant PDAC cells [94].

## 6. PDT in Combination with Other Therapies

The combination of PDT with classical chemotherapy drugs has shown promise for synergy and to potentially reduce the chemotherapy dose and associated systemic toxicity [95,96,97]. Chemoresistance in cancer is associated in part with the hallmark characteristic of resisting cell death by increased antiapoptotic signaling [98]. PDT has been shown to target the anti-apoptotic proteins such as BCL-2, thus tipping the balance toward pro-apoptotic signaling and making cancer cells more responsive to chemotherapy [99]. Gemcitabine, although it only provides marginal survival benefit, has remained the primary treatment for advanced pancreatic cancer [96,100]. It has been shown that combination of PDT with low-dose gemcitabine significantly reduced the volume of the pancreatic tumor without any adverse effect in vivo [101]. One study investigated the possibility of enhancing oxaliplatin efficacy using benzoporphyrin derivative-mediated (BPD, verteporfin)–PDT in 3D culture models [102]. The results from this study indicate that the combination of these therapies was significantly more effective compared to either therapy alone. This was due to the distinct cytotoxic mechanisms of these therapies. While oxaliplatin induced DNA damage, BPD–PDT mainly targeted mitochondrial membrane and as a result lacked any overlap in toxicity of the chemotherapy agent. This is an important point to consider since it provides better disease management to healthy tissue. The study also showed that combining two mechanistically distinct therapies did not guarantee the enhancement in the therapy response and emphasized the need for physiologically relevant models to assess combinations. Similar treatment effects were observed in other cancer models that suggest in general PDT synergizes with platinum-based chemotherapies [103,104,105,106]. Moreover, PDT and chemotherapy not only have distinct mechanisms of action within the cell, but they also target different cells within the tumor. The results from a 3D model of PDAC showed that while chemotherapy was able to decrease the live volume of the spheroid, it had almost no effect on the invading cells. The same study showed that the opposite result was true for PDT, where invading cells died the most after treatment [92]. Additionally, it has been shown that PDT is able to disrupt adherent junctions between cancer cells [107]. This could be potentially significant if PDT is administrated prior to chemotherapy, enhancing paracellular drug transport into nodules, as well as by depleting surrounding stroma as discussed earlier. Furthermore, use of PDT as a pre-treatment in which the light-based production of ROS by design is not sufficient to surpass the cytotoxicity threshold but does elicit biological response. This approach, which has been termed photodynamic priming (PDP), has been shown to overcome chemotherapy resistance by combination with vitamin D3 receptor activation in the pancreatic tumor microenvironment (TME) [108].

In addition to interacting with classical chemotherapies, PDT may also play an inherently complementary role with other light-based approaches. Photothermal therapy (PTT) is a therapeutic technique that uses photothermal agents (usually gold) to convert the absorbed light into heat, which induces cancer cell death [109,110]. This method by itself or in combination with other therapies have been used for pancreatic cancer. The combination of PDT and PTT provides a promising strategy to enhance therapeutic efficiency in PDAC [111]. Although PTT by itself is unable to eliminate all cancer cells in the tumor due to constant heat lost by circulating blood, when combined with PDT, it can compensate (for the reduced efficacy) of oxygen-dependent PDT in the hypoxic tumor [112,113].

An inherent challenge in PDT treatment of solid tumors is the limitation of light penetration in biological tissue. Sonodynamic therapy (SDT) is another ROS-dependent strategy that works based on the synergistic interaction of drugs and ultrasound. Due to the relatively low tissue attenuation coefficient of ultrasound, it can penetrate deeply into the tissue [114]. Yet another ROS-dependent strategy is chemodynamic therapy (CDT), which works based on H_2_O_2_ conversion into toxic hydroxyl radicals (OH) and leads to cell apoptosis [115]. Combination of PDT and SDT or CDT are relatively new approaches that can overcome the hypoxia in PDAC and as a result enhance the generation of ROS in pancreatic cancer [116,117,118].

PDT is also conducive to combination with radiation therapy (RT), with which it shares similar dosimetry and overlapping communities of practitioners and medical physicists. For example, it has been shown that Cerenkov radiation produced by high-energy X-rays passing through tissue can activate PS [119,120,121]. Photosensitizers for PDT may also serve as radiosensitizers, potentially providing improved radiation delivery to target tissue while reducing overall radiation dose. The combination of PDT and RT has also been explored through the innovative combination of radioluminescent nanoparticles which are excited during RT with deep-tissue-penetrating X-rays, producing luminescence which in turn activates conjugated PS to achieve a low-dose PDT effect in deep tissue [122]. A recent study on 3D pancreatic cancer coculture models investigated whether PDT synergizes with RT when combined in the absence of nanoparticles and showed the beneficial effect of PDT on RT efficacy [123]. This study suggests the potential of nanoscintillator-induced PDT as another strategy for deep-tissue treatment, where both therapies are simultaneously activated by the ionizing radiations.

Immune checkpoint blockade (ICB) therapy has demonstrated promising responses in several types of cancers. A recent study has surveyed the effect of PDT, RT, and PTT on stimulating a number of immune modulatory effects [124]. In pancreatic cancer, however, ICB therapy has shown limited benefits, since its TME has been shown to be highly immunosuppressive [125]. The immunologic effects of PDT, such as releasing antigens and immunogenic factors from generated ROS, make PDT an interesting option for combination with immunotherapy in tumors [126]. In a mouse model study, they were able to boost T-cell activation and overcome adaptive immune resistance by combining PDT with BRD4 inhibitor (BID4i) [125].

## 7. Emerging and Future Directions

Given the noted role of stroma in PDAC progression and as a barrier to drug delivery, the concept of therapeutically targeting PDAC stroma has emerged as a potentially important strategy. As discussed here, multiple lines of evidence suggest that PDT may have some role to play in this context. In a 3D PDAC co-culture model, it was shown that while the nodules in the presence of fibroblasts were more chemoresistant, PDT response was enhanced in the presence of fibroblasts [78]. Another study on PDAC cocultured with fibroblasts in monolayer showed non significantly higher PDAC cell death in the presence of non-activated fibroblasts [127]. Furthermore, a study demonstrated that PDT not only depleted stromal fibroblasts, but also interrupted crosstalk with stromal signaling partners that gave rise to enhance tumor survival [128]. Combined with additional evidence that PDT can induce breakdown of ECM components [129], these preclinical results collectively indicate a potential role for PDT in depleting cellular and non-cellular PDAC stromal components to enhance subsequent drug delivery. Clinically this scenario could be leveraged by activating the PS at early timepoints following delivery when initial stromal accumulation is highest.

In consideration of how PDT may play a role in the next generation of cancer therapeutics, the opportunity for interactions with RNA medicine emerges as a potentially exciting avenue of investigation. In recent years, RNA medicine has demonstrated exciting potential for a wide variety of diseases, including, specifically, PDAC, through targeting of microRNAs (miRNAs) [130,131,132,133]. MiRNAs are small (~18–25 nucleotides) non-coding RNAs that can bind target mRNAs in a sequence-specific fashion to induce post-transcriptional downregulation. Several studies have already identified miRNAs with significantly altered expression between normal pancreas and PDAC tissues; among them, miR-21, miR-196a, and miR-196b, which are strongly correlated with decreased survival [134,135]. In a mouse model of miR-21 over-expression, it has been revealed that the mice develop tumors in tissue where miR-21 is over-expressed, and that these tumors depend on the continued expression of miR-21 for survival [136]. These lines of evidence highlight the role of ‘oncomiR addiction’ in regulating key pathways promoting tumor growth, survival, and chemoresistance [137]. Furthermore, miR-21 depletion using a nanoparticle to carry an anti-miRNA inhibitor also inhibits organoid growth, suggesting the potential of this approach as a therapeutic strategy [130]. At the same time, this approach opens new potential avenues for synergy with PDT. Inhibition of miR-21 has been shown to increase levels of the pro-apoptotic factor BAX, while PDT with verteporfin is known to target anti-apoptotic factors BCL-2 and BCL-XL [138,139]. Similarly, targeting of another PDAC onco-miR, miR-196b, has also been shown to promote resistance to late-stage apoptosis in PDAC cells [140]. Combination of PDT with selective therapeutic inhibition of these oncomiRs could synergistically increase the Bax/Bcl-2 ratio (pro-/anti-apoptotic) in PDAC cells and tip the balance toward apoptosis in these otherwise stubbornly drug-resistant cells. In addition to synergizing at the molecular level, combination with PDT could also enhance delivery of RNA medicines through depletion of stromal components. As noted above, the notoriously dense fibrotic stroma in PDAC is problematic for delivery of virtually all therapeutic agents, and this may be especially true for RNA medicine. While various anti-miRNA strategies have been discussed for the past decade, a lack of adequate delivery to most disease tissues has restricted current therapeutic uses to liver and kidney disease. Collectively, these observations point to the potential benefits of leveraging nanoparticle delivery systems that could simultaneous carry light-activated agents for PDT to target PDAC stroma for enhanced delivery of RNA medicine agents, while at the same time priming tumor cells for enhanced biological response to these therapies.

## 8. Conclusions

Collectively, the literature points to multiple significant roles for PDT in the clinical management of PDAC. The clinical studies discussed above have established the techniques for light delivery which enable PDT as a primary treatment for locally advanced PDAC, which could play a key role especially for disease which is unresectable. However, biological responses to PDT may also synergize with systemic therapies as part of a complete treatment strategy. At the same time, active and ongoing research continues to reveal new roles for PDT and its potential to interact with other promising strategies that are just beginning to emerge.
